# Dramatic response to entrectinib in a rare glioneuronal tumor harboring an *NTRK2* fusion

**DOI:** 10.1093/oncolo/oyaf418

**Published:** 2025-12-15

**Authors:** Firas Akrout, Henri Bogumil, Mohamed Dehmani Yedeas, Philipp Sievers, Mehdi Touat, Sameh Achoura

**Affiliations:** Department of Neurosurgery, Military Hospital of Tunis, Tunis, Tunisia; Faculty of Medicine of Tunis, University of Tunis El Manar, Tunis, Tunisia; Department of Neuropathology, Institute of Pathology, University Hospital Heidelberg, Heidelberg, Germany; Clinical Cooperation Unit Neuropathology, German Consortium for Translational Cancer Research (DKTK), German Cancer Research Center (DKFZ), Heidelberg, Germany; Department of Neurosurgery, Military Hospital of Tunis, Tunis, Tunisia; Faculty of Medicine of Tunis, University of Tunis El Manar, Tunis, Tunisia; Department of Neuropathology, Institute of Pathology, University Hospital Heidelberg, Heidelberg, Germany; Clinical Cooperation Unit Neuropathology, German Consortium for Translational Cancer Research (DKTK), German Cancer Research Center (DKFZ), Heidelberg, Germany; Inserm, CNRS, UMR S 1127, Institut du Cerveau et de la Moelle épinière, ICM, Sorbonne Université, Paris, France; AP-HP, Hôpitaux Universitaires La Pitié Salpêtrière—Charles Foix, Service de Neurologie 2-Mazarin, Paris, France; Department of Neurosurgery, Military Hospital of Tunis, Tunis, Tunisia; Faculty of Medicine of Tunis, University of Tunis El Manar, Tunis, Tunisia

**Keywords:** Glioneuronal tumor, GTAKA, *NTRK* fusion, Entrectinib, DNA methylation profiling

## Abstract

Glioneuronal tumors are rare CNS neoplasms that can exhibit overlapping histological features with embryonal tumors, posing diagnostic and therapeutic challenges. We report a case of a 19-year-old Tunisian woman with a large right frontal tumor and contralateral extension. Initial partial resection suggested CNS neuroblastoma, and the patient underwent chemoradiotherapy with temporary disease control. Upon progression, a second partial resection was followed by DNA methylation profiling, which reclassified the tumor as a glioneuronal tumor with *ATRX* alteration, kinase fusion, and anaplastic features (GTAKA), harboring a *KANK1::NTRK2* fusion. Entrectinib therapy was initiated, leading to a complete radiological response at 14 months, with marked clinical improvement and no serious adverse effects. This case highlights the essential role of DNA methylation profiling in resolving diagnostic ambiguity and guiding targeted treatment in CNS tumors. It further supports the potential efficacy of entrectinib in *NTRK* fusion–positive glioneuronal tumors.

Key points:

- DNA methylation profiling established the definitive GTAKA diagnosis, corrected the initial CNS neuroblastoma misclassification, and identified actionable *NTRK* fusions, that guided an effective targeted therapy.
- This case represents the first documented clinical response to TRK inhibition in a glioneuronal tumor with *ATRX* alteration, kinase fusion, and anaplastic features (GTAKA).
- TRK inhibitors may provide rapid and clinically meaningful benefit even in *NTRK* fusion–positive tumors with substantial residual volume.

## Introduction

Glioneuronal tumors represent a heterogeneous group of central nervous system (CNS) neoplasms, with emerging molecular classifications reshaping diagnostic paradigms and therapeutic approaches. Among these, Glioneuronal Tumor with *ATRX* Alteration, Kinase Fusion, and Anaplastic Features (GTAKA) has recently been delineated as a distinct molecular entity. It is defined by a characteristic DNA methylation signature and recurrent gene fusions, predominantly involving the *NTRK* gene family.^1^ These oncogenic fusions are key drivers of tumor growth and survival, making TRK proteins attractive targets for precision therapy. Entrectinib, a CNS-penetrant tyrosine kinase inhibitor with potent activity against TRK, ROS1, and ALK fusion proteins, has demonstrated efficacy in *NTRK*-rearranged solid tumors such as non-small cell lung cancer and secretory carcinomas.^2^ However, its application in primary CNS tumors, particularly rare glioneuronal subtypes like GTAKA, remains understudied. We present a case of *NTRK* fusion-driven GTAKA with sustained and rapid clinical and radiological response to entrectinib, illustrating how molecular profiling can identify actionable targets and expand therapeutic options for aggressive glioneuronal tumors.

## Patient story

**Figure 1.**
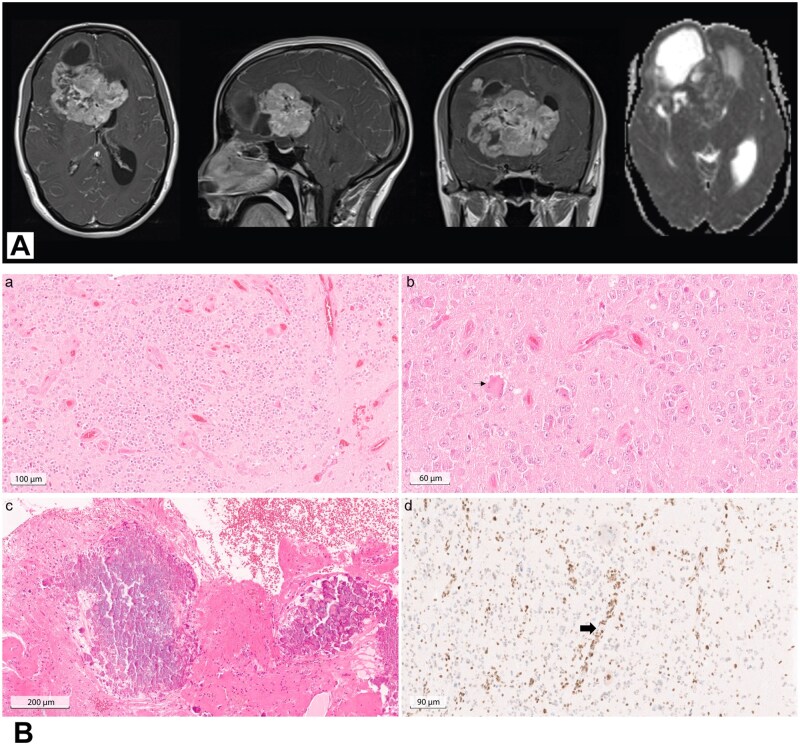
(A) Preoperative cranial MRI of the patient (from left to right: axial, coronal and sagittal T1-weighted with gadolinium injection, axial apparent diffusion coefficient). There is a solid intra-axial mass within the right frontal lobe with contralateral involvement. The enhancing component of the lesion shows diffusion restriction. (B) HE slides of the primary tumor showed a cell dense glioneuronal tumor with microvascular proliferation (a, scale bar 100 µm). Multinuclear cells (b, small arrow, scale bar 60 µm) as well as calcification (c, scale bar 200 µm) could be observed. ATRX immunohistochemistry (d, scale bar 90 µm) revealed near complete loss of nuclear staining in tumor cells in comparison to non-neoplastic cells such as blood vessels (large arrow).

The patient, a 19-year-old Tunisian woman with no past medical history, presented with symptoms of raised intracranial pressure. Neurological examination was unremarkable except for a frontal syndrome characterized by disinhibited behavior. MRI showed a large supratentorial lesion mainly located in the right frontal lobe with contralateral involvement. The enhancing component of the lesion exhibited diffusion restriction. MR spectroscopy demonstrated a clear increase in choline with a decrease of the N-acetyl aspartate (NAA) peak (Figure 1(A)). A partial resection was performed. The initial histological diagnosis, based on morphological and immunohistochemical studies performed in two different expert neuropathology centers, was a primary CNS neuroblastoma (WHO 2016 grade IV).^3^ The tumor proliferation showed high cellularity and consisted of neurocytic-like cells, which were mostly positive for Olig2 and synaptophysin and partially positive for Lin28 and Neu-N. MRI at 3 months postoperatively showed a neoplastic residue in the tumor bed and disease progression. Based on the original diagnosis, three cycles of EP (cisplatin (100 mg/m^2^/d) and etoposide (120 mg/m^2^/d)) were administered. Chemotherapy was interrupted due to grade 3 neutropenia after the second and third courses. Concomitantly, the patient received whole brain irradiation (30.6 Gy) with a boost to the initial tumor bed (23.4 Gy). Follow-up MRI scans showed a significant partial response, with an estimated 59.2% reduction in tumor size. After a period of disease stability for 26 months from therapy discontinuation, the patient developed symptoms of elevated intracranial pressure and cognitive impairment. Imaging revealed significant progression of the residual tumor and associated hydrocephalus, prompting a repeat partial resection, placement of a ventriculoperitoneal shunt, and a subsequent re-evaluation of the primary tumor’s pathology.

## Molecular tumor board

Tumor samples were referred for an expert opinion to Heidelberg. Histological analyses showed a cell-dense glioneuronal tumor with elevated mitotic activity and microvascular proliferation (Figure 1(B)). Adjacent CNS tissue revealed hemosiderin pigment, fresh hemorrhages and focal microinfarction.

**Figure 2.**
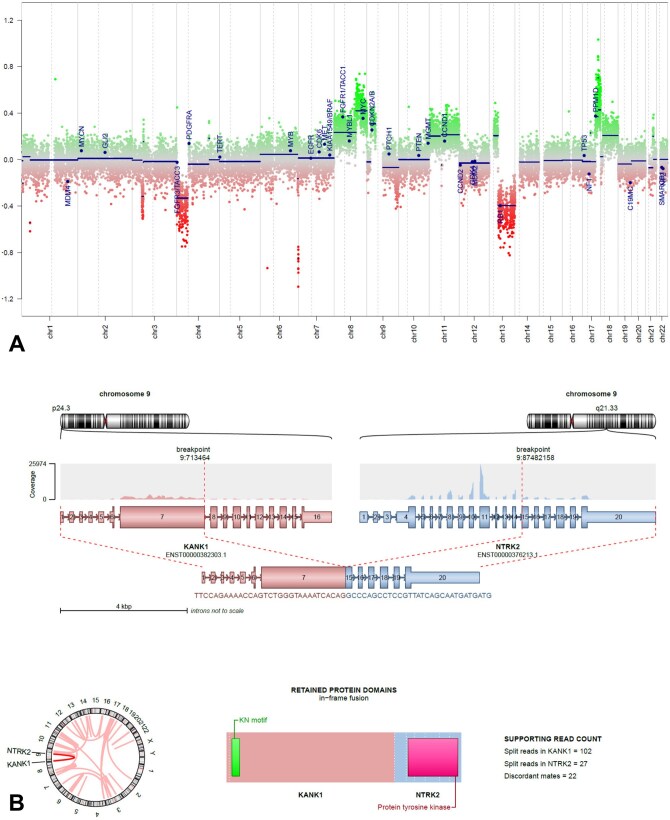
(A) Copy number variation profile generated from DNA methylation data shows several chromosomal gains and losses. (B) Visualization of the detected *KANK1::NTRK2* fusion including chromosomal breakpoints and involved exons of both fusion partners.

For further classification, DNA methylation analysis was performed using Illumina XX methylome array, with analysis using the diagnostic Heidelberg pipeline.^4^ The methylation data showed a match (0,90) with the methylation class super-family Adult Type Diffuse Gliomas with the highest score (0,89) for the methylation class “Glioneuronal tumor with *ATRX* alteration, kinase fusion and anaplastic features (novel)”. Copy number variation profile revealed several chromosomal gains (chromosomes 8, 11 and 17) and losses (chromosomes 4 and 13) as well as a balanced CDKN2A/B status (Figure 2(A)). MGMT promoter was predicted unmethylated. Consistent with the results of DNA methylation analysis, ATRX immunohistochemistry was performed (mouse monoclonal, clone BSB-108, dilution 1:2000, Bio SB, Santa Barbara, CA, USA) and showed a near complete loss of ATRX nuclear expression in tumor cell nuclei compared to non-neoplastic cells (Figure 1(B)).

As tumors of the methylation class “Glioneuronal tumor with *ATRX* alteration, kinase fusion and anaplastic features (novel)” usually harbor kinase fusions,^1^ RNA sequencing was performed using a previously described diagnostic pipeline.^5^ An oncogenic in-frame *KANK1::NTRK2* fusion was detected with preserved protein tyrosine kinase domain of *NTRK2* (Figure 2(B)). In conclusion of histology and molecular analyses, an integrated diagnosis of “Glioneuronal tumor with *ATRX* alteration, kinase fusion and anaplastic features (GTAKA)” was established.

To further investigate the ATRX staining, a DNA panel sequencing was performed using a previously described diagnostic workflow.^6^ Analyses showed two missense variants in *ATRX*, which were not yet described in the literature (Table 1), fitting to the diagnosis.

**Table 1.** Summary of *ATRX* gene variants identified by targeted DNA panel sequencing.

Gene	Consequence	Transcript of variant	Allele frequency	Allele depth	Depth of sequencing	Classification according to ClinGen-CGC-VICC Guidelines^7^	Reference genome
***ASXL1***	missense variant	NM_015338.6: c.2105T>C | p. Leu702Pro | chr20: g.32434817T>C	50.41	1169	2319	VUS	hg38
***ATRX***	missense variant	NM_000489.6: c.3392G>A | p. Arg1131Lys | chrX: g.77681864C>T	20.8	57	274	VUS	hg38
***ATRX***	missense variant	NM_000489.6: c.3557G>A | p. Arg1186Lys | chrX: g.77681699C>T	19.67	155	788	VUS	hg38
***MET***	missense variant	NM_000245.4: c.3443G>T | p. Arg1148Leu | chr7: g.116778878G>T	50.35	284	564	VUS	hg38
***NOTCH1***	missense variant	NM_017617.5: c.2296G>A | p. Gly766Ser | chr9: g.136513449C>T	32.91	968	2941	VUS	hg38
***NOTCH2***	missense variant	NM_024408.4: c.4996G>A | p. Val1666Ile | chr1: g.119922642C>T	45.05	387	859	VUS	hg38
***PLXNB1***	missense variant	NM_001130082.3: c.2215T>C | p. Trp739Arg | chr3: g.48420071A>G	75.93	975	1284	VUS	hg38
***POLD1***	missense variant	NM_002691.4: c.3317C>T | p. Ala1106Val | chr19: g.50417940C>T	46.65	746	1599	VUS	hg38
***SETD2***	missense variant	NM_014159.7: c.5476C>T | p. Arg1826Cys | chr3: g.47084304G>A	46.31	257	555	VUS	hg38
***SMARCD3***	missense variant	n.151275131G>T | chr7: g.151275131G>T	48.5	663	1367	VUS	hg38
***ZMYM3***	missense variant	NM_201599.3: c.1879C>T | p. Arg627Trp | chrX: g.71248258G>A	43.9	587	1337	VUS	hg38

## Patient update

**Figure 3.**
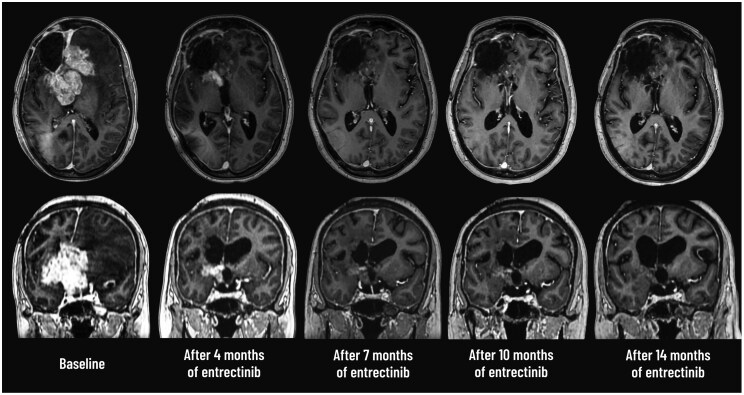
Radiological evolution of the tumor under entrectinib therapy. Axial, and coronal post-contrast T1-weighted MRI sequences obtained at baseline, and after 4, 7, 10 and 14 months of entrectinib treatment (from left to right). A marked and progressive reduction in the volume of the enhancing tumor is observed over time, indicating a sustained radiological response.

Given the identification of an oncogenic *NTRK2* fusion, the patient was initiated on the selective TRK inhibitor entrectinib at a daily dose of 600 mg. Within the first three months of therapy, she achieved full restoration of cognitive function and maintained a Karnofsky performance status of 90%, allowing her to resume her university studies and reclaim independent daily activities without assistance. Successive follow-up MRIs at 4, 7, and 10 months showed a progressive reduction in tumor volume, resulting in complete radiological regression at 14 months and confirming a durable response to entrectinib (Figure 3). The treatment was well tolerated, demonstrating a favorable safety profile. Aside from a brief episode of dysgeusia and weight gain, no other toxicities were observed, and laboratory parameters remained unremarkable. At the time of this report, the overall treatment duration and follow-up are 14 months and 8 days.

## Discussion

Accurate classification of CNS neoplasms remains challenging in neuropathology, particularly when tumors exhibit overlapping histological and immunophenotypic features. As illustrated by our case, glioneuronal tumors such as GTAKA can histologically be misclassified as CNS neuroblastoma, or vice versa, owing to shared morphological characteristics (small round blue cell appearance) and neuronal marker expression (synaptophysin, NeuN).^8–10^ This diagnostic ambiguity carries profound clinical consequences: embryonal tumors like neuroblastoma conventionally require aggressive multimodal chemotherapy, while *NTRK* fusion–driven glioneuronal tumors may derive durable benefit from targeted inhibitors such as entrectinib. Misclassification can therefore expose patients to unnecessary toxicities from cytotoxic regimens while delaying access to effective biology‑driven therapies.

This dilemma underscores the imperative of DNA methylation profiling in CNS tumors with ambiguous or heterogeneous histology, particularly those displaying mixed glial/neural or glial/embryonal components. By comparing a tumor’s genome-wide methylation pattern to established reference profiles, this method accurately identifies the tumor type, determines its WHO grade, and reveals targetable molecular alterations, as shown in our case. Our findings align with a growing consensus that methylation analysis should be integrated as a first‑line diagnostic modality for histologically equivocal CNS neoplasms, thereby enabling tailored, precision‑based management.^11^

Entrectinib is a potent, orally bioavailable, CNS-penetrant small-molecule inhibitor designed to selectively target tyrosine receptor kinases involved in oncogenic gene fusions. Specifically, it inhibits the tropomyosin receptor kinases TRKA, TRKB, and TRKC (encoded by *NTRK1*, *NTRK2*, and *NTRK3*, respectively), as well as ROS1 and ALK fusion proteins. These oncogenic fusions drive tumorigenesis by promoting cell proliferation and survival. In August 2019, entrectinib received FDA approval for adult and pediatric patients ≥12 years with *NTRK* fusion-positive solid tumors and for *ROS1*-positive non-small cell lung cancer, representing a milestone in the adoption of molecularly guided cancer therapies. Other TRK inhibitors include the first-in-class agent larotrectinib, approved in 2018, and several next-generation inhibitors currently in development, such as selitrectinib (LOXO-195), repotrectinib (TPX-0005), and taletrectinib (DS-6051b), which aim to overcome resistance to first-generation agents.

Entrectinib has demonstrated robust efficacy across a range of *NTRK* fusion–positive solid tumors, including non-small cell lung cancer, secretory breast carcinoma, and salivary gland tumors.^2^ Its ability to penetrate the blood–brain barrier makes it particularly relevant for the treatment of primary CNS tumors, which often present therapeutic challenges due to limited drug delivery to the CNS.^12^ In the phase 1/2 STARTRK‑NG trial, entrectinib demonstrated rapid and durable activity against pediatric intracranial and extracranial solid tumors harboring *NTRK1/2/3* or *ROS1* fusions.^13^

While clinical benefit from *NTRK* inhibitors in gliomas and glioneuronal tumors with *NTRK* fusions is documented in several case reports and small series, prospective data remain limited, and the target holds an ESCAT IIB score, pending validation of efficacy in larger clinical trials or registries.^14^^,^^15^ The literature specifically addressing *NTRK* fusion–positive glioneuronal tumors treated with TRK inhibitors is particularly sparse, with only four cases published to date. Two cases, harboring *ARHGEF2–NTRK1* and *BCAN–NTRK1* fusions, were treated with entrectinib and achieved partial responses, with follow-up durations of 9 and 6 months, respectively.^16^^,^^17^ The other two cases, involving *STRN1–NTRK2* and *MEF2D–NTRK1* fusions, were treated with larotrectinib and demonstrated complete responses maintained at 11 and 13 months of follow-up, respectively.^18^^,^^19^ Notably, all previously reported cases involved minimal residual tumor volume at the time of TRK inhibitor initiation, whereas our patient received entrectinib in the context of a substantial 6 cm residual tumor, highlighting the potential of TRK inhibition even in cases with high tumor volume. Our findings further support the importance of testing for *NTRK* fusions and the potential therapeutic value of targeted treatment in this context.

To our knowledge, this is the first reported case of a GTAKA showing a rapid and profound clinical and radiological response to entrectinib. The near-complete regression achieved within 16 weeks underscores the rapid therapeutic efficacy of TRK inhibition, even in tumors with high initial volume. This case reinforces the critical role of early molecular profiling in identifying actionable alterations such as *NTRK* fusions and highlights the promise of precision medicine in the management of rare and aggressive CNS tumors.

## Data Availability

All data are included in this article. Additional details are available from the corresponding author upon reasonable request.

## CRediT statement

Firas Akrout (Conceptualization, Formal analysis, Investigation, Writing—original draft), Henri BOGUMIL (Investigation, Writing—original draft), Mohamed Dehmani Yedeas (Investigation), Philipp Sievers (Investigation, Supervision, Writing—review & editing), and Mehdi Touat (Supervision, Validation), Sameh Achoura(Investigation, Supervision, Validation).
